# Follicular Viability and Histological Alterations after Auto-transplantation of Dog Ovaries by Experimentally Inducing Blood Sinus on Stomach

**Published:** 2011-03-21

**Authors:** Hazhir Khoram, Alireza Najafpour, Mazdak Razi

**Affiliations:** 1 Clinical Science Department, Faculty of Veterinary Medicine, Islamic Azad University, Urmia Branch, Urmia, Iran; 2 Comparative Histology and Embryology, Faculty of Veterinary Medicine, Urmia University, Urmia, Iran

**Keywords:** Ovarian, Transplantation, Sexual Hormone, Canine

## Abstract

**Background:**

Currently, chemotherapy and radiotherapy are considered most effective methods for cancer treatment, however these strategies often result in fertility problems. A favorable alternative to prevent fertility loss in cancer patients is the cryopreservation and transplantation of sexual tissues (ovaries and/or testes). There is a low rate of fertilization following cryopreservation of ovaries prior to implantation. Therefore, in our opinion, this low rate is caused by instable blood flow during organ transplantation. Thus, this study researches a canine ovarian model that focuses on direct exposure of ovaries with blood in an experimentally induced sinus-like cavity. We implanted this tissue on the muscular layer of the stomach, which is its most vascularized region.

**Materials and Methods:**

Ovarian transplantation was conducted on T1 animals (n=5), bilateral ovariectomy was performed on T2 animals (n=5), unilateral ovariectomy was conducted on T3 cases and animals in the control-sham group (n=5) did not undergo ovariectomy or transplantation.

**Results:**

All isotransplanted ovaries survived. Ovaries resumed follicular growth and revascularization. Transplanted ovaries contained 75%-76% of survived small follicles (pre antral) after 60 days. The ovarian granulosa cells showed considerable resistance against ischemia. After day 30 no statistically significant differences in the level of estradiol and progesterone were observed between T1 animals and the T3 group. T1 animals showed considerably high levels of progesterone and estradiol in comparison to T2 cases.

**Conclusion:**

This study showed that using blood sinus method for ovarian isotransplantation helps ovarian tissue to survive from post implantation ischemia which confirms with normal follicles presentation and intact endocrine function of the implanted ovaries.

## Introduction

Chemotherapy and radiologic treatments are important, effective methods for the treatment of cancer. Meanwhile, oncology treatments are associated with long-term infertility effects. Following oncological treatment, remarkable toxicity occurs in ovarian tissue which in turn leads to a severe loss in the ovarian follicular bank, finally resulting in fertility problems (1, 2). Thus, the collection and storage of oocytes and embryos (cryopreservation) are the most predominant protocols in order to protect ovaries from the adverse effects of radiological and/or chemotherapeutic techniques. On the other hand, effective oocyte cryopreservation needs hormonal stimulation in order to increase oocyte numbers. Such stimulations delay the initiation of anticancer therapy and might directly cause growth of progressive tumors which are dependent on hormonal alteration (3, 4). Furthermore, only post pubertal humans and/or animals can be considered for oocytes or ovarian cryopreservation; it is not an appropriate method for very young cancer patients (5).

In order to protect the ovaries as well the ovarian follicular banks from the side effects of the above mentioned therapeutic methods, numerous research has been conducted to remove ovarian tissue, at least for a therapeutic period. The first attempt for ovarian transplantation in animals and humans dates from the 19th century. According to an early report, following transplantation of a mice ovary on the ovarian bursa several neonates were born (6). In another study, transplantation of ovarian tissue to the upper arm resulted in a successful live birth in the rhesus monkey that used in vitro fertilization for egg growth (7). Lastly, several reports have shown successful results in the transplantation of ovarian fragments to different regions (8, 9), however after organ transplantation, observations demonstrated that remarkable follicular damage occurred because of severe ischemia post transplantation (10,11). Further observations have demonstrated unsatisfactory results; estradiol secretion continues for 6 to 8 months and often disappears after 1 year. Finally the ovarian physiological bioactivities will be limited to 3 to 4 months (12). Thus, attempts focused on cryopreserving the organ before implantation on an appropriate region. However the findings were discouraging. Following cryopreservation of the fragments, the crystal formation can not be ignored and at the same time, the blood flow can not be guarded, thus in turn it can lead to ovarian and follicular necrosis which results in poor quality oocytes (12-14). In addition, findings have shown that transplantation of the cryopreserved ovaries yielded a very low pregnancy rate (about 0.2%) (15).

To avoid ischemic injury due to unstable blood flow, attempts have recently been made to use vascular anastomosis. The vascular anastomosis of the ovary is remarkably smaller in comparison to the other organs and transplantation with vascular anastomosis requires high level techniques known as super micro-surgery (16). Although micro-surgery is very useful, this technique needs highly equipped hospitals. Thus, at the first step of the current work we tried to dissect the ovaries by rapid and careful ovariectomy and auto-transplant the fragmented ovaries to the anterior-stomach capsule by creating a sac like cavity (blood sinus). Light microscopic analyses were conducted to investigate ovarian histological structure and follicular survival. Finally, the hormonal alterations were measured to understand any probable changes in gonadal hormones. Our present study breaks new ground and focuses on blood flow in transplantation, which may be lead to new findings.

## Materials and Methods

### Experimental design

In this study all experiments which conducted on animals were in accordance with the guidance of Ethical Committee for research on laboratory Animals of Urmia University.

The present study used 20 mature female dogs obtained from West Azerbaijan, Urmia (Iran ecotypes). We divided the animals into the following groups. Test group 1 (T1=5) underwent ovarian tissue transplantation. Test group 2 (T2=5) underwent bilateral ovariectomies. In test group 3 (T3=5) unilateral ovariectomies were performed with transplants. The control-sham group (CS=5) did not have ovariectomies or transplants. In order to prevent oxidative stress, vitamin E (150 mg/ kg) was administrated directly to the serum during surgery and given intra muscularly, 2 days after surgery, every 24 hours for a period of 2 weeks.

### Ovarian transplantation

Ovaries were divided in two equal pieces from the middle line of the ovarian tissue. Half of the ovaries were implanted in subserosa of the greater curvature area on the muscular layer of the stomach wall. The ovary was grafted intra-muscularly by inducing hemorrhage in the region and closing the stomach seromuscular flap with absorbable suture material in two layers in the form of a saclike cavity in order to directly expose the implanted ovaries to blood in the experimentally created blood sinus.

### Histomorphological analyses

On day 60 following surgery, the ovaries were removed and fixed in formaldehyde acetic solution (IFAA, Germany) for 4 weeks. Ultimately, they were dissected free from per-ovarian tissues. Samples were processed through paraffin embedding and serially cut with a rotary microtome, and stained with the hematoxylin and eosin technique.

### Follicular characteristics and numbers

Follicular morphology was examined by microscope (×400). All follicles in the test and controlsham groups were counted and recorded depending on their sizes. Follicles were classified as 100 and 101-200 μm (small or pre-antral follicles). Normal follicles had a complete layer of flattened granulosa cells, oocytes with cytoplasm, and a normal nucleus. Abnormal follicles were classified as follows: cytoplasmic damage, pyknotic nucleus, and combination of damaged nucleus and cytoplasm. Follicular number was estimated by counting follicles in all slides (17).

### Assessment of ovarian arteries and veins

Light microscopic investigation of the ovarian medullar, cortical arteries and veins showed the general histological structure of the tissue vessels. The histological characteristics for normal and abnormal arteries and veins were investigated (18). In order to identify the endothelial cells reorganized in the ovarian inner and outer medulla, cortex and to evaluate vessels’ fibrotic and muscular integrity, a special staining for blood vessel endothelial cells, collagen fibers and smooth muscle cells was conducted. Endothelial cells, a major component of the vascular inner layer, were stained by horseradish peroxidase conjugated Bandeiraea simplicifolia BS-1 isolectin (Sigma Co.) and visualized with 3-amino-9-ethylcarbazole (AEC; Sigma) (17, 19).

### Serum sampling and hormonal analyses

Blood samples from corresponding animals were collected directly from the heart on days 10, 20, 30, 40, 50 and 60 after surgery, centrifuged (3000 rpm/5 minutes) and subjected to assays of serum progesterone and estradiol. Progesterone and estradiol were assessed by electrochemilunescence.

### Statistical analysis

All results are presented as mean ± SD. Differences between quantitative histological and hematological data were analyzed with two-way ANOVA, followed by Bonferroni test, using Graph Pad Prism 4.00,. P<0.05 was considered significant. Correlation between total follicular number with survived follicles were analyzed on an Indigo-2 O2 work station (Silicon Graphics, Mountain View, CA) using Matlab (MathWorks, Inc., Natick, MA).

## Results

### Ovarian follicular viability and number per one ovary

**Fig 1:**
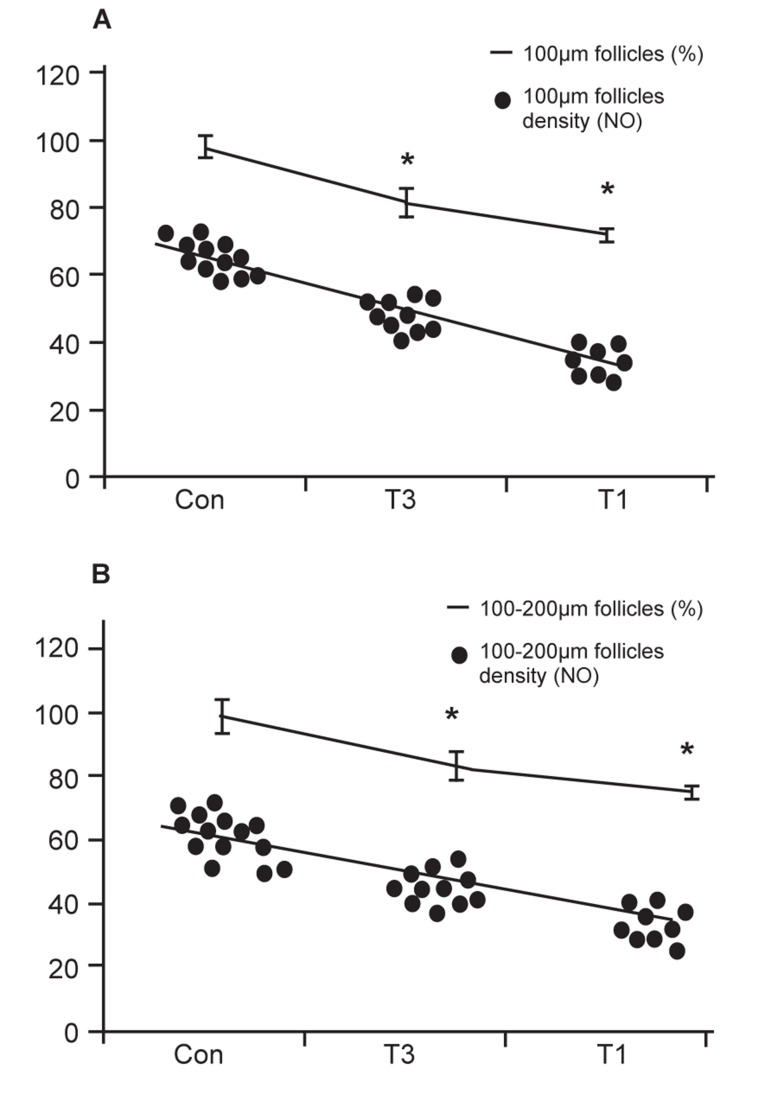
Correlation between total follicular number (density) with percent of follicles that survived. Black spots are represent total follicular density. Smooth lines illustrate the percentage of follicles that survived. Total follicular number positively correlated with the percentage of 100 μm survived follicles, r2=0.068; p≤0.05 and 101-200 μm survived follicles r2= 0.79; p≤0.05.

Histological observations demonstrated that in T1 animals total follicular numbers (per one ovary) decreased in comparison to T3 and controlsham animals. Analysis of correlation between total follicular numbers with follicular viability per one ovary showed that the number of total follicles that survived in T2 ovaries approximated T3 viable follicles. The data for follicular viability and number (per one ovary) are presented in figure 1.

### Histological examination of small size follicles

Light microscopic investigations revealed that the transplanted ovaries exhibited more damage in the oocyte cytoplasm, nucleus and/or combination of cytoplasm and nucleus (p≤0.05) in comparison to the intact ovaries in the T3 and control-sham groups (Table 1).

**Table 1:** Comparisons of <100 and 101-200 µm follicular oocyte damage between control-sham T1 and T3 groups


Groups	100 µm intact follicles (%)	Cytoplasmdamage (%)	Nucleus damage (%)	Cytoplasm &nucleus damage (%)

**Control-sham**	98.09 ± 1.43	11.8 ± 1.30	1.95 ± 0.61	1.48 ± 0.39
**T1 group**	78.17 ± 1.32*	14.6 ± 1.67*	2.79 ± 0.14*a	1.92 ± 0.16
**T3 group**	81.60 ± 2.07*	14.4 ± 1.14*	2.08 ± 0.05*a’	1.89± 0.23

**Groups**	**101-200 µm Intact follicles (%)**	**Cytoplasm damage (%)**	**Nucleus damage (%)**	**Cytoplasm & nucleus damage (%)**

**Control-sham **	98.80 ± 1.64	7.00 ± 0.81	1.22 ± 0.43	0.77 ± 0.52
**T1 group**	79.75 ± 2.06*b	11.25 ± 0.95*c	2.55 ± 0.32*d	1.67 ± 0.45*e
**T3 group**	83.87 ± 0.95*b’	8.75 ± 0.50*c’	2.02 ± 0.05*d’	1.16 ± 0.21e’


Stars indicate significant differences (p≤0.05) between T1 and T3 animals with control-sham in the same column. Different letters and superscripts in the same column indicate significant differences (p≤0.05) between T1 and T3 animals. All data are presented as mean ± SD

**Fig 2:**
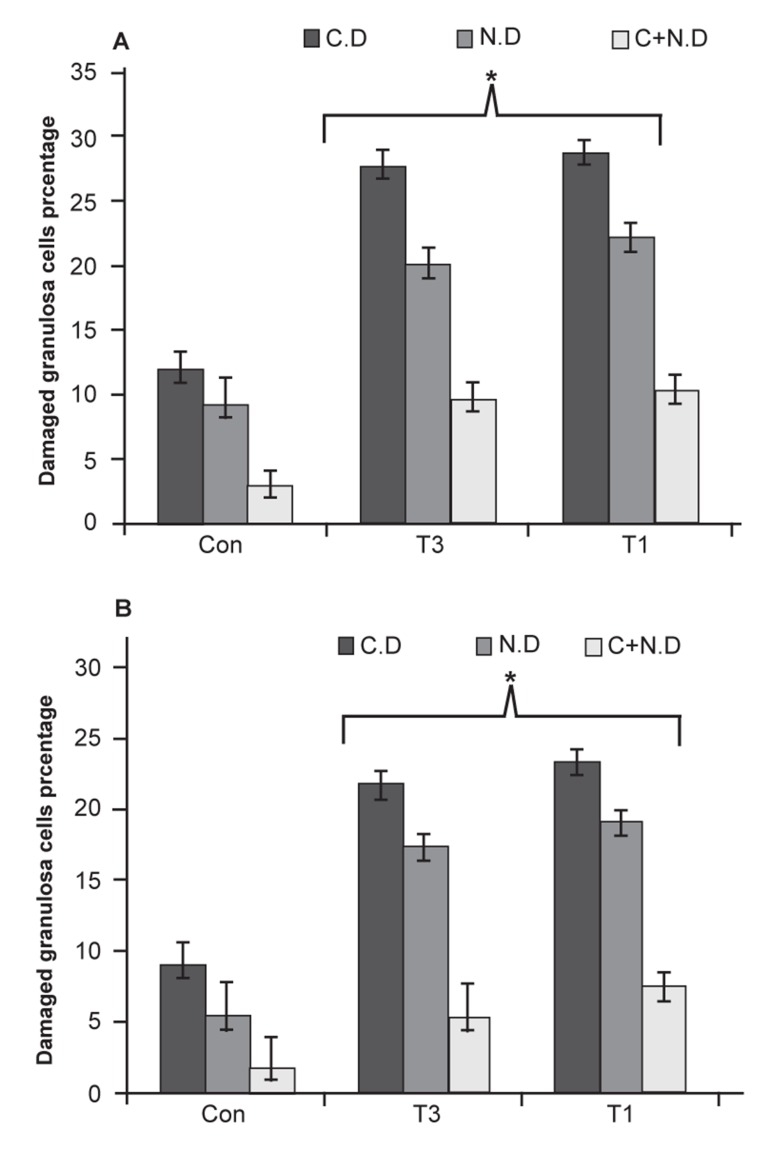
Mean percentage of cytoplasmic damage (C.D), nuclear damage (N.D) and combined cytoplasmic+nuclear damage of granulosa cells. Stars indicate significant differences (p≤0.05) between T1 and T3 groups with the control-sham group. There are no statistically significant differences (p≥0.05) between T1 and T3 animals. All data are presented as mean ± SD.

In contrast to oocytes, analysis of the same damages in granulosa cells showed no significant differences (p≥0.05) between T1 and T3 groups (Fig 2).

### Transplantation toxicity on ovarian parenchyma and vessels

Light microscopic analyses illustrated that ovaries in the T1 group underwent light necrosis, particularly in regions far from the blood vessels. Necrotic cells were located in sub-capsular regions and/or adjacent with the capsule. The parenchyma close to new generated blood vessels manifested with approximately normal histological appearance. The intact remaining ovaries in the T3 and control-sham groups had no necrotic parenchyma. Blood vessels of the T1 cases exhibited no fractures in any of the animals but very low bloated vascular muscle cells, endothelial cell damage and hypertrophy as detected by light microscopic analyses (Table 2).

Reorganized endothelial cells were present in the inner and outer medulla of the transplanted ovaries (Figes3).

There were several histologically normal veins and arterioles in the medulla and cortical regions of the implanted ovaries. There were no statistically significant differences (p≥0.05) between histologically normal veins and arterioles of the T1 and T3 groups (Fig 4).

### Hematological examination of hormones

Blood serum analyses illustrated that the serum level of estradiol decreased in both T1 and T3 animals. Meanwhile, after day 40 there were no significant (p≥0.05) differences between T1 and T3 serum estradiol levels. T2 animals maintained constant levels of estradiol (20.78 ± 1.40) during 60 days.

**Table 2:** Histological assessment of ovarian medulla and cortex arteries and veins endothelial bloating (EB), hypertrophy (EH) and degeneration (ED) in control-sham, T1 and T3 groups.


Ovaries	Medullar artery			Medullar vei		
	BD	EH	BE	BD	EH	BE

**Control-sham**	0	0	0	0	0	0
**T1 group**	12.33 ± 1.03	10.50 ± 1.04*	9.61 ± 0.49	8.75 ± 1.25	8.25 ± 0.95*	8.00 ± 0.81
**T3 group**	11.50 ± 1.37	9.11 ± 0.77*	9.81 ± 1.00	8.10 ± 1.52	8.12 ± 0.77*	7.63 ± 0.44

**Ovaries**	**Cortical vein**			**Cortical artery**		
	**BD**	**EH**	**BE**	**BD**	**EH**	**BE**

**Control-sham**	0	0	0	0	0	0
**T1 group**	8.78 ± 0.42	6.39 ± 0.57	6.00 ± 0.63	5.37 ± 0.62	5.60 ± 0.61*	4.21 ± 0.49
**T3 group**	8.38±0.48	6.09±0.79	5.16±0.98	4.95±0.60	5.18±0.76 *	4.26±0.60


Stars indicate significant differences (p≤0.05) between data in the same column. All data are presented as mean±SD.

**Fig 3:**
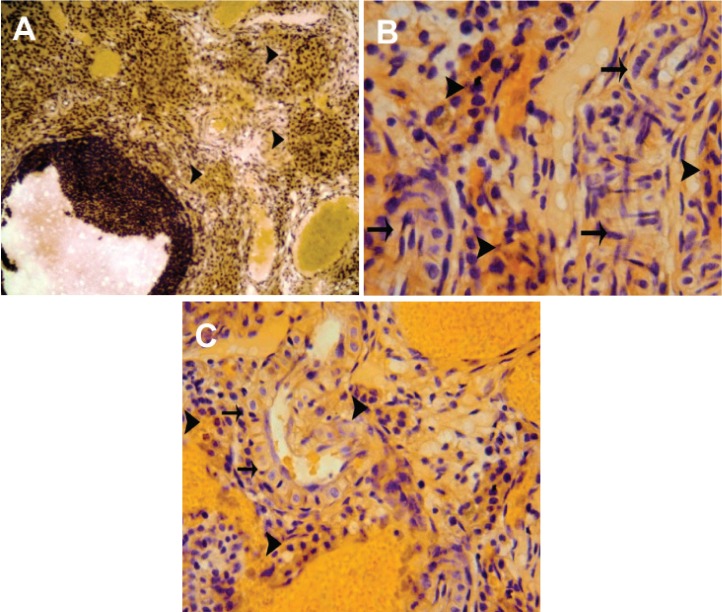
Histological architecture from the transplanted ovary. A. Low magnification. Note the dark brown sites (arrow heads) close to the recovered blood vessels showing newly organized endothelial cells in order to generate new blood vessels. B. High magnification from outer medulla of the transplanted ovary. Note the dark brown stained endothelial cells aggregated abundantly close to light brown stained cells showing recovered endothelial cells (arrows). C. High magnification from inner medulla, dark brown stained endothelial cells (arrow heads) located adjacent to the endothelial cells with heterogeneous cytoplasm (arrows), endothelial cell staining (A, ×100; B and C, ×400).

**Fig 4:**
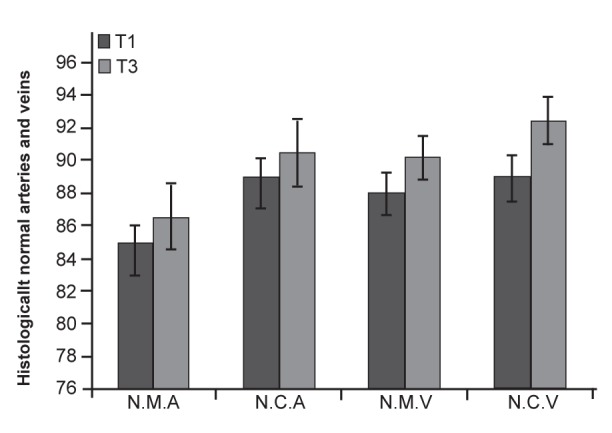
Histological comparison of normal arterioles and veins in the medullar and cortical regions of the ovaries in T1 and T3 animals. Data are presented in percent (%). Note: N.M.A, normal medullar arteriole; N.C.A, normal cortical arteriole; N.M.V, normal medullar vein and N.C.V, normal cortical vein. There were no significant differences (p≥0.05) between data evaluated for T1 and T3. All values are presented as mean ± SD.

**Fig 5:**
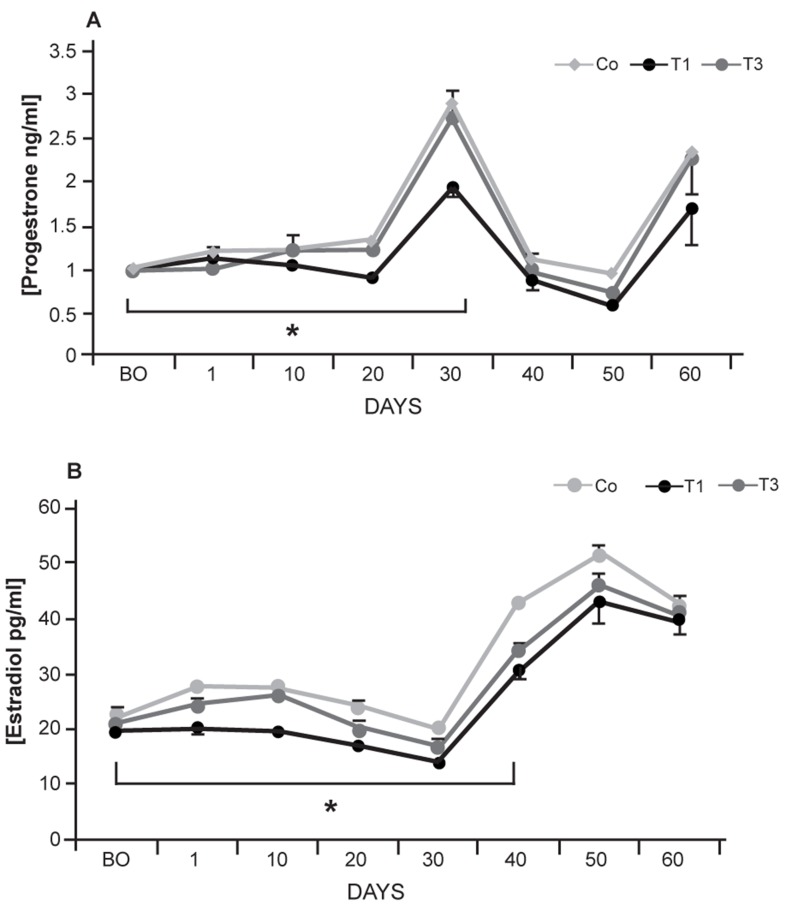
Blood level of progesterone (A) and estradiol (B). Stars indicate significant differences (p≤0.05) for progesterone levels on days 1 to 30 and estradiols level on days 1 to 40 between T1 and T3 groups. There were no considerable differences (p≤0.05) between data for T1 and T3 progesterone and estradiol levels after days 30 and 40. There were remarkable differences (p≤0.05) between data for both T1 and T3 groups with control-sham animals during all days. All values are presented as mean ± SD.

Although the serum level of progesterone was constant the first day after transplantation, a statistically significant (p≤0.05) decrease occurred until day 30 in both T1 and T3 groups. After day 30, the blood level of progesterone began to increase in both T1 and T3 groups, which was not statistically different (p≥0.05). Data for blood estradiol and progesterone levels are presented in Figures 5-A and 5-B. T2 group showed an approximately constant level of progesterone during 60 days (0.177 ± 0.008). A comparison of estradiol and progesterone blood levels in the T1 and T3 groups with control-sham animals showed lower serum levels of these hormones in the experimental groups compared with the controlsham group. Meanwhile, after days 30 and 40, serum levels of progesterone and estradiol began to stabilize.

## Discussion

In recent decades there have been striking advances in the treatment of cancer by using chemotherapy and/or radiotherapy methods. As survival and cure rates rise, the focus is turning to the late effects of treatments, of which the loss of fertility and gonadal failure seem to be very importance points. Nowadays various options exist such as oocyte and sperm cryopreservation in adults (18). Secondly, embryo banking for females and even in vitro oocyte maturation are considered alternative preserving methods for the protection of fertility in humans and animals (19). However some of the above mentioned strategies are not applicable in clinical settings. Most importantly, none of the mentioned protocols are practical for pre-pubertal ages, and there are increasing number of girls and boys who are cancer survivors (20). There are reports of successful transplantation of ovarian tissue from a number of species including laboratory rodents (21), farm animals (22) and monkeys (23). According to Israely et al., organ transplantation to the muscular region has shown better results in neovascularization (17). Thus, in the present study we transplanted ovaries onto the muscular layer of the stomach. Our observations revealed that neovascularization occurred remarkably and the ovaries maintained and survived histologically after implantation. The rich blood supply within the muscles provided superior graft reception in the current animals.

Although total follicular numbers decreased in T1 animals, an evaluation of the percentage of follicles that survived in correlation with total follicular number showed that more than half of the total follicular population survived after transplantation in T1 animals. According to animal and human preliminary studies, the key factor responsible for follicular survival seems to be post-graft ischemia. As the process of revascularization can take more than a day to complete, thus tissue ischemia can be a problem for implants (17, 24-26). Follicular survival spatially relates to the presence of pericytes, endothelial cells and/or neovasculation in the area of the graft (26, 27). Following transplantation, the ovarian cortex shows better follicular survival which is probably due to a sufficient blood supply. According to several reports, mRNA expression of two angiogenic factors, vascular endothelial growth factor (VEGF) and transforming growth factor b-1 (TGFb-1), is up-regulated mainly at the ovarian cortex 48 hours after transplantation (26). Our histological studies have shown that 75-76% of the follicles survived implant ischemia. This situation suggests that the current method may reduce the degenerative effect of post implant ischemia by direct and close adjustment of the blood with the graft during first days after transplantation. On the other hand, special staining for analyzing the histological healthiness of blood vessels in different regions of the transplanted ovaries illustrated that after 60 days, huge reorganization of the endothelial and smooth muscle cells occurred in transplanted ovaries and lately generated endothelial cells infiltrated between newly oriented ovarian parenchyma. Observations demonstrated light damages in the deep medullar region of the T1 animal’s ovaries, which can be accompanied by regression of the pericytes and smooth muscle cells of the blood vessels. This impairment was symptomatic of an insufficient blood supply. Although some areas from the medullar region of the grafts showed necrosis, the superficial medullary regions adjacent to the neovasculated vessels were normal. The cells in this area were probably nourished by the exudates from the recovered and/or reorganized vessels.

Comparing endothelial cell damage and bloating between T1 and T3 animals showed no considerable differences between these two groups. This situation suggests that the intact ovary in the T3 animals began to have compensatory revascularization following unilateral ovariectomy and therefore some alterations manifested in older endothelial cells. On the other hand, the transplanted ovaries of the T1 group showed the abovementioned alterations, the same as T3 animals, but these alterations were not statistically considerable in comparison to the T3 group. May be after implantation the ovaries started to reorganize the flow of blood by compensatory revascularization. Furthermore, the environmental condition (post implanting side effects) could be considered another reason for structural changes in T1 animal ovarian vessels.

It is well known that the ovary is responsible for female hormonal (estrogen and progesterone) secretion and fertility. Once ovarian function disrupts, women and female animals experience sexual difficulties and will probably have serious problems in developing secondary sexual characteristics. The ovarian transplantation method used in the present study is expected to protect endocrine function because of the preservation of ovarian granulosa cells. Resumption of the menstrual cycle after transplantation is regarded as fertility in gynecology but this understanding is not completely correct. In transplantation with no vascular anastomosis, the menstrual cycle may resume because some oocytes and granulosa cells remain alive. However, the quality of the follicles can severely spoil due to ischemic injuries. Although the remaining poor quality oocytes and granulosa cells maintain the sexual cycle, fertility is expected to be low (24, 27). According to our hematologic investigations, estradiol and progesterone levels decreased in comparison to T3 and control-sham animals until day 30. However after day 30, the blood level of these hormones increased with no significant differences manifested between T1, T3 and control- sham groups. Animals in group T1 showed considerably higher blood concentration for estradiol and progesterone in comparison to the T2 animals, showing resumption of menstrual cycle in transplanted animals.

## Conclusion

Here we report that survival of the ovaries after implantation depends on blood support and our study showed that direct adjustment of the blood during the first days after transplantation could help ovarian tissue survive from post implantation ischemia. Furthermore our findings showed that this method (experimentally inducing a blood sinus around the transplanted ovaries) is the type of reconstructive surgery for simultaneously conserving neovasculation by reorganization of blood vessels endothelial, muscular cells and fibrotic structure associated with protecting endocrine function of the grafted ovaries.

## References

[1] Meirow D, Nugent D (2001). The effects of radiotherapy and chemotherapy on female reproduction. Hum Reprod Update.

[2] Torrents E, Boiso I, Barri PN, Veiga A (2003). Applications of ovarian tissue transplantation in experimental biology and medicine. Hum Reprod update.

[3] Nicholson HS, Byrne J (1993). Fertility and pregnancy after treatment for cancer during childhood or adolescence. Cancer.

[4] Kim SS, Battaglia DE, Soules MR (2001). The future of human ovarian cryopreservation and transplantation: fertility and beyond. Fertil Steril.

[5] Carrel A, Guthrie CC (1906). Technique de la transplantation homoplastique de l'ovaire. C R Seances Soc Biol.

[6] Parrott DMV (1960). The fertility of mice with orthotopic ovarian grafts derived from frozen tissue. J Reprod Fertil.

[7] Lee DM, Yeoman RR, Battaglia DE, Stouffer RL, Zelinski- Wooten MB, Fanton JW (2004). Live birth after ovarian tissue transplant. Nature.

[8] Hilders CG, Baranski AG, Peters L, Ramkhelawan A, Trimbos JB (2004). Successful human ovarian autotransplantation to the upper arm. Cancer.

[9] Donnez J, Dolmans MM, Demylle D, Jadoul P, Pirard C, Squifflet J (2004). Livebirth after orthotopic transplantation of cryopreserved ovarian tissue. Lancet.

[10] Meirow D (2000). Reproduction post-chemotherapy in young cancer patients. Mol Cell Endocrinol.

[11] Marconi G, Quintana R, Rueda-Leverone NG, Vighi S (1997). Accidental ovarian autograft after a laparoscopic surgery: case report. Fertil Steril.

[12] Andersen CY, Rosendahl M, Byskov AG, Loft A, Ottosen C, Dueholm M (2008). Two successful pregnancies following autotransplantation of frozen thawed ovarian tissue. Hum Reprod.

[13] Imhof M, Bergmeister H, Lipovac M, Rudas M, Hofstetter G, Huber J (2006). Orthotopic microvascular reanastomosis of whole cryopreserved ovine ovaries resulting in pregnancy and live birth. Fertil Steril.

[14] Koshima I (2008). Atypical arteriole anastomoses for fingertip replantations under digital block. J Plast Reconstr Aesthet Surg.

[15] Mihara M, Nakagawa T, Noguchi S, Due M, Yieu SA (2009). MRI, magnetic resonance influenced organ freezing method under magnetic field. ACSC.

[16] Courbiere B, Caquant L, Mazoyer C, Franck M, Lornage J, Salle B (2009). Difficulties improving ovarian functional recovery by microvascular transplantation and whole ovary vitrifi cation. Fertil Steril.

[17] Israely T, Dafni H, Granot D, Nevo N, Tsafriri A, Neeman M (2003). Vascular remodeling and angiogenesis in ectopic ovarian transplants: A crucial role of pericytes and vascular smooth muscle cells in maintenance of ovarian grafts. Biol Reprod.

[18] Witherington R, Black JB, Karow AM (1977). Jr.Semen cryopreservation: an update. J Urol.

[19] Fabbri R, Porcu E, Marsella T, Rocchetta G, Venturoli S, Flamigni C (2001). Human oocyte cryopreservation: new perspectives regarding oocyte survival. Hum Reprod.

[20] Aziz NM (2002). Cancer survivorship research: challenge and opportunity. J Nutr.

[21] Candy CJ, Wood MJ, Whittingham DG (2000). Restoration of a normal reproductive lifespan after grafting of cryopreserved mouse ovaries. Hum Reprod.

[22] Gosden RG, Baird DT, Wade JC, Webb R (1994). Restoration of fertility to oophorectomized sheep by ovarian autografts stored at-196 degrees C. Hum Reprod.

[23] Schnorr J, Oehninger S, Toner J, Hsiu J, Lanzendorf S, Williams R (2002). Functional studies of subcutaneous ovarian transplants in non-human primates: steroidogenesis, endometrial development, ovulation, menstrual patterns and gamete morphology. Hum Reprod.

[24] Shinohara T, Inoue K, Ogonuki N, Kanatsu-Shinohara M, Miki H, Nakata K (2002). Birth of offspring following transplantation of cryopreserved immature testicular pieces and in-vitro microinsemination. Hum Reprod.

[25] Baird DT, Campbell BK, de Souza C, Telfer E (2004). .Long term ovarian function in sheep after ovariectomy and autotransplantation of cryopreserved cortical strips. Eur J Obstet Gynecol Reprod Biol.

[26] Dissen GA, Lara HE, Fahrenbach WH, Costa ME, Ojeda SR (1994). Immature rat ovaries become revascularized rapidly after autotransplantation and show a gonadotropin-dependent increase in angiogenic factor gene expression. Endocrinology.

[27] Neeman M (2000). Preclinical MRI experience in imaging angiogenesis.. Cancer Metastasis Rev.

